# Forecasting secular variation using physics-informed neural networks for IGRF-14

**DOI:** 10.1186/s40623-026-02427-6

**Published:** 2026-04-28

**Authors:** N. Shakespeare-Rees, P. W. Livermore, C. J. Davies, H. F. Rogers, C. D. Beggan, W. J. Brown, C. C. Finlay

**Affiliations:** 1 School of Earth and Environment, University of Leeds, Woodhouse, Leeds, LS2 9JT UK; 2 British Geological Survey, Edinburgh, UK; 3 DTU Space, Technical University of Denmark, Copenhagen, Denmark

**Keywords:** Physics Informed Neural Networks (PINNs), Regional Inversion Methods, Secular Variation, Outer Core Flow, IGRF-14

## Abstract

In response to the call for candidate models for the 14th generation of the International Geomagnetic Reference Field (IGRF) by the Geomagnetic Field Modeling Working Group (V-MOD) of the International Association of Geomagnetism and Aeronomy (IAGA), we present the University of Leeds candidate model for the forecast of the average Secular Variation (SV) for 2025–2030. Our approach consists of inverting the geomagnetic field model CHAOS−7.18 using Physics-Informed Neural Networks to produce two global mesh-free models from (i) a mosaic of independent regional flows and (ii) a single global flow representation. The magnetic field is then advected under the assumption of steady core flow over a 5-year period, and the average SV over 5 years is taken to construct the forecast. We validate our approach using hindcasts for the IGRF-13 time period (2020–2025) and benchmark our methodology against the inferred SV from CHAOS−7.18. Our field models constructed from regional flows show reduced RMS misfit relative to the field from the CHAOS−7.18 model at each yearly timestep, compared to the other candidate models from IGRF-13 in the hindcast, both at the Core Mantle Boundary and at the Earth’s Surface. We then present our IGRF-14 candidate forecast for the period 2025–2030, derived from the regional method, and discuss possible improvements to this method for future IGRF submissions.

## Introduction

The Earth’s main magnetic field, powered by fluid motions in the Earth’s outer core that drive a self-sustaining dynamo (Bullard 1950), changes over a range of timescales. The observed timescales span years to millennia (Constable and Constable 2023) and temporal variations in the geomagnetic field on timescales of years to millennia are termed Secular Variation (SV) (Jackson and Finlay 2015). The geomagnetic field has been measured by networks of ground-based observatories since 1837 (Macmillan 2007), and has been further supported by continuous satellite measurements since 1999 (Friis-Christensen et al. 2006; Olsen and Stavros 2011; Olsen and Floberghagen 2018; Olsen et al. 2020; Zhang 2023; Jiang et al. 2024). Temporal variations in the geomagnetic field is significant for both industry and scientific research across various fields, including navigation, satellite operations, and space weather hazards.

To try to understand and predict changes in the geomagnetic field, models, such as the International Geomagnetic Reference Field (IGRF), are created. Commonly, these models are constructed in spectral space with global basis functions, such as spherical harmonics, constrained by measurements from satellites, observatories and other surveys. A benefit to using spherical harmonic global basis functions is the ability to analytically downward continue the field model to the Core–Mantle boundary (CMB). The IGRF is a collaborative effort overseen by the Division V Geomagnetic Field Modelling Working Group (V-MOD) of the International Association of Geomagnetism and Aeronomy (IAGA), which is updated every 5 years. Intervening epochs are forecast through an average SV model, itself derived from multiple candidate models. The previous edition of IGRF is the 13th iteration (Alken et al. 2021b), which contains a definitive main field model for 2015.0, a main field model for epoch 2020.0, and a predicted secular variation for 2020.0 to 2025.0.

The construction of the IGRF has driven advances in geomagnetic field modelling, evidenced by the variety of methods used to model the field. Responding to previous IGRF calls, candidate methodologies for secular variation have included constructing models from ground and satellite measurements (Alken et al. 2021a; Finlay et al. 2020; Rother et al. 2021; Huder et al. 2020), data assimilation techniques (Fournier et al. 2021; Minami et al. 2020; Sanchez et al. 2020), Kalman-filter-based models (Tangborn et al. 2021; Baerenzung et al. 2020), interpolation (Petrov and Bondar 2021), diffusion-based models (Maurits et al. 2020a), bootstrapping (Javier et al. 2020) and spectral analysis (Wardinski et al. 2020). All of these models, except for the diffusion-based model presented by Metman et al. (2020b), assume that the observed SV is produced by advection of the fluid in the outer core.

A frequently used assumption when inverting the geomagnetic field to map flows at the edge of the outer core is to assume that on sufficiently short timescales, magnetic diffusion can be considered negligible—this is the *frozen flux* assumption presented in Roberts and Scott (1965). Under this simplification, the magnetic field lines can be thought to be ‘frozen’ within moving liquid packets at the surface of the outer core, effectively making the change in the magnetic field a tracer of the flow (Bloxham 1991). Non-uniqueness is further minimised by assuming large scale flow, wherein small scale flow structures are penalised (Bloxham 1988). Flow features inferred from previous studies include an eccentric, anticyclonic, planetary gyre (Pais and Jault 2008), changes in the azimuthal flow direction in areas in the equatorial region (Whaler et al. 2022; Jinfeng et al. 2024), and a localised jet under the Bering Strait (Livermore et al. 2017).

For our IGRF-14 SV forecast, we employ a steady advection-only core flow approach. A candidate model for IGRF-13 based on the steady-flow advection of the outer core flow was presented by Brown et al. (2021), with two candidate models of IGRF-12 also being based on steady core flow advection (Hamilton et al. 2015). However, in contrast to prior approaches, constructed using global basis functions, we implement two novel aspects based on neural networks: (i) a global model created by a mosaic of regional flows individually and independently inferred by a fit to a local realisation of a global geomagnetic field model, and (ii) a global model, inferred through a single global fit to a field model. Our regional approach provides an independent method to check global flow analyses, providing complementary information for the global candidate models, as a regularised large-scale global inversion may act to smooth out important local flow features. In addition, in a model dependent on global basis functions, any point on the CMB will be influenced non-locally by the SV at all the other locations on the CMB, but in a regional flow inversion the same point only depends on the other points in that local region. This is important, because, although the quality and coverage of geomagnetic measurements from satellites and ground-based observatories are generally good, satellite data are more susceptible to contamination from external fields in auroral regions, while the spatial distribution of the observatory network remains highly uneven. Our regional methodology can probe the reliability of local features in these areas; we can also be sure that flow maps constructed in non-polar regions are not contaminated by polar data with significant unmodelled non-internal signal.

Local core flow inversions have been attempted in previous studies. Rogers (2022) studied regional variations using spherical Slepian functions. This technique produced better separation of SV at the Earth’s surface compared to spherical harmonics but reliable local flow separations and SV separations at the CMB were not achieved. Schwaiger et al. (2023) presented a local core flow inversion methodology based on pointwise inversion, which was able to reproduce the main features found in global core flow studies, but it was found that additional smoothing was required to prevent unreliable re-construction of small-scale flows. They also found that their results heavily depended on what prior they used to reduce the non-uniqueness. Our approach infers regional flows through the use of Physics-informed neural networks (PINNs), a class of machine learning models, described in Shakespeare-Rees et al. (2025).

Machine learning refers to a collection of statistical techniques used to leverage data to complete a task, allowing the user to extract knowledge and draw inferences (Jordan and Mitchell 2015). Neural networks (NN), a machine learning architecture, consist of layers of artificial neurons that can process information and learn complex representations of information. PINNs, first proposed in Raissi and Karniadakis (2019), are a class of neural network that integrate mathematical descriptions of physical laws as soft constraints to solve forward and inverse problems. PINNs have been used in a diverse range of fields, such as fluid mechanics (Raissi et al. 2020), medicine (Arzani et al. 2021), nuclear physics (Schiassi et al. 2022) and seismology (Chen et al. 2022). PINNs have been recently used in core flow and length of day (LOD) analysis by Jinfeng et al. (2024), albeit only in a global flow inversion framework.

The rest of this paper is organised as follows: in Sect. 2, we describe our core flow inversion methodology using PINNs, and then detail our advection scheme which produces the SV forecast in Sect. 3. In Sect. 4, we show results for hindcasting, benchmarking our hindcast models against IGRF-13 candidate models, and then present our candidate SV forecast for IGRF-14 in Sect. 5. Finally, we discuss our results and areas of improvement for our methodology in Sect. 6.

## Core flow inversion using PINNs

Modelling the Earth’s core as a sphere described using spherical coordinates $(r, \theta , \phi )$, we assume that the flow, $\boldsymbol{u}$, within the sphere obeys a non-penetration boundary condition at the edge of the core with vanishing radial flow, $u_r = 0$, at the CMB. The radial magnetic field, $B_r$, can then be related to the horizontal fluid motion at the edge of the core via the radial component of the induction equation, which assuming frozen flux, can be written as

1 $$\begin{aligned} \frac{\partial B_r}{\partial t} = -\nabla _H \cdot (\boldsymbol{u}B_r), \end{aligned}$$

in which $\boldsymbol{u} = [0, u_\theta , u_\phi ]$ is the flow that is sought in the inversion methodology, and $\nabla _H$ is the horizontal gradient operator.

In spherical coordinates, the total flow $\boldsymbol{u}$ can be decomposed into toroidal and poloidal flows:

2 $$\begin{aligned} {\boldsymbol{u}} = \nabla \times T(\theta ,\phi ) \textbf{r} + \nabla _H (r P(\theta ,\phi )), \end{aligned}$$

whose horizontal components can be written (Holme 2015):

3 $$\begin{aligned} & \boldsymbol{u}_T = \left( \frac{1}{\sin {\theta }}\frac{\partial \textit{T}}{\partial \phi }, -\frac{\partial \textit{T}}{\partial \theta }\right) , \end{aligned}$$

4 $$\begin{aligned} & \boldsymbol{u}_P = \left( \frac{\partial \textit{P}}{\partial \theta }, \frac{1}{\sin {\theta }}\frac{\partial \textit{P}}{\partial \phi }\right) . \end{aligned}$$

**Fig. 1:**
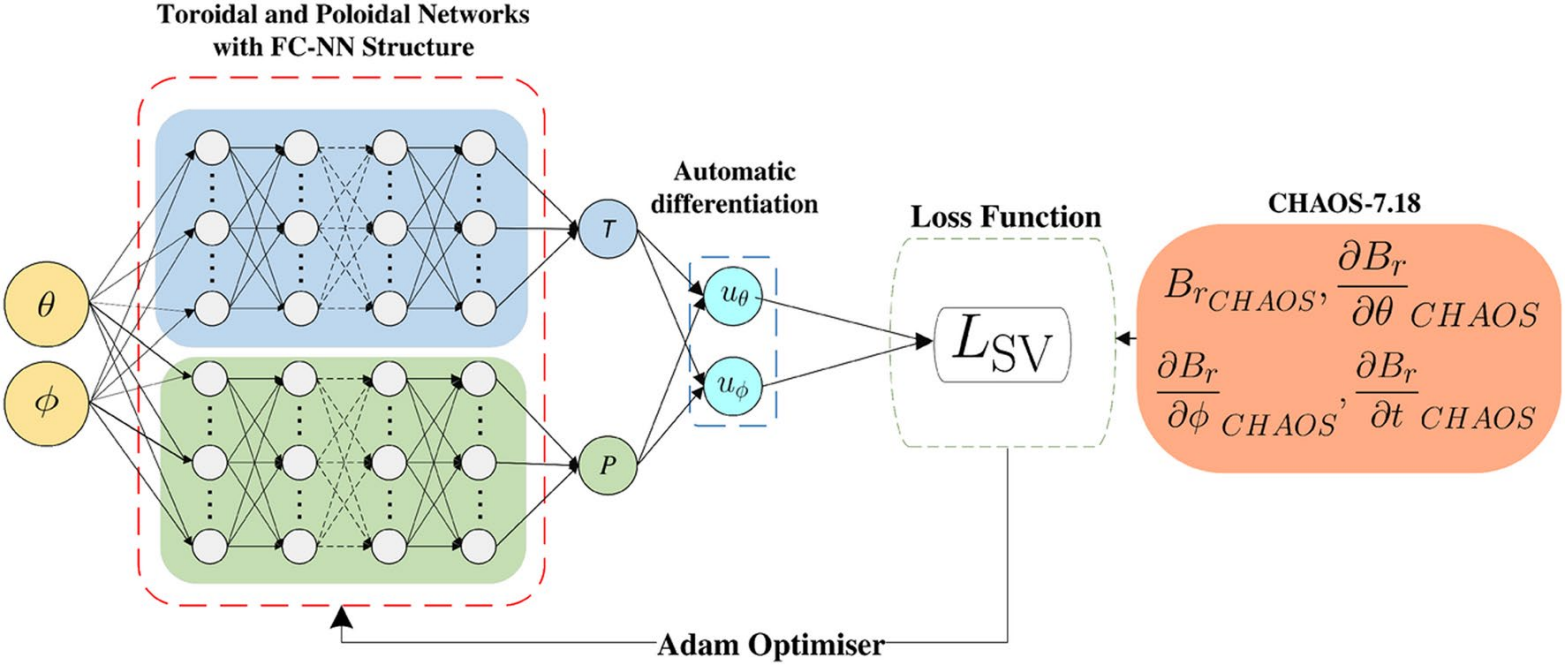
Schematic of the PINN used in this study, where $\theta , \phi$ are co-latitude and longitude, *T* and *P* are the toroidal and poloidal scalars, and $u_\theta$ and $u_\phi$ are the components of the horizontal flow. Yellow indicates inputs to the network, the two parallel FC–NNs are blue and green, loss function in white, data inputs to the loss function in orange, and the outputs are cyan

Therefore, we seek toroidal (*T*) and poloidal (*P*) scalar functions which define the flow in our flow inversion. Our inversion methodology consists of two fully connected neural networks (FC-NNs) working in parallel: one to describe the toroidal scalar function *T* and the other to describe the poloidal scalar function *P*. A schematic for this is shown in Fig. 1. FC-NNs are composed of layers of nodes, including an input layer, one or more hidden layers, and an output layer (Jordan and Mitchell 2015). Each node is interconnected with others and is associated with a specific weight and bias. The output of a node is calculated by applying a non-linear function—known as the activation function—to the weighted input. During a process known as training, the network adjusts its weights and biases to improve performance (Goodfellow et al. 2016). Once the network weights are determined, the flows are then described using Eqs. (3) and (4).

The networks are trained using the optimisation method Adam (Kingma and Ba 2017), which updates both networks concurrently. The PINN receives a grid of points defined by ($\theta , \phi$) at a radius of 3485 km. It then analytically computes the toroidal and poloidal scalar value at each of those points, then calculates the spatial derivatives of these scalars using Pytorch’s built-in differentiation engine (Adam et al. 2017). These derivatives are then used in Eqs. 3 and 4 to produce $u_\theta , u_\phi$. The loss term in the PINNs used for this submission consists of the MSE (Mean Squared Error) between the SV recreated by the PINN-based flows and the SV from the CHAOS−7.18 model:

5 $$\begin{aligned} L_\text {SV}= \frac{1}{N} {{\sum \limits _{i}^{N}}}\left[ \frac{\partial B_r}{\partial t}_{PINN} - \frac{\partial B_r}{\partial t}_{CHAOS} \right] ^{2}\bigg |_{\theta _i, \phi _i}, \end{aligned}$$

where *N* is the number of points, and the SV calculated from the horizontal flows is

6 $$\begin{aligned} \frac{\partial B_r}{\partial t}_{PINN} = &-\frac{1}{r} \left( \frac{u_\phi }{\sin \theta } \frac{\partial B_r}{\partial \phi }_{CHAOS} + u_\theta \frac{\partial B_r}{\partial \theta }_{CHAOS}\right) \\ & - B_{r_{CHAOS}}(\nabla _{H} \cdot \boldsymbol{u}) . \end{aligned}$$

The Adam optimizer back-propagates the loss through the network, updating weights and biases to minimize the loss via gradient descent over 5,000 iterations. However, empirical results show that $L_{SV}$ as a function of the model parameters exhibits complex behaviour, leading the optimiser to only converge to local minima (Shakespeare-Rees Naomi et al. 2025). We, therefore, choose multiple different seeds—equivalent to varying initial weights—for independent training, from which we select the model that achieves the lowest loss. Given that our dataset is of order 1000–10,000 points—modest by machine learning standards—we are able to use all the data in each iteration of training. The PINN is constructed using SciANN (Haghighat and Juanes 2021), a TensorFlow/Keras wrapper for physics-informed machine learning, with straightforward implementation. We highlight that other approaches to constructing PINNs are available, such as the PyTorch approach described in Shakespeare-Rees et al. (2025).

To optimise training, a number of hyper-parameters need to be chosen. The learning rate—governing how rapidly the weights change with each iteration—was chosen to be the standard value of $10^{-3}$, as it was found empirically that this resulted in the lowest SV misfit. Our choice of activation function was swish (Ramachandran et al. 2017), due to its better performance when compared to other activation functions, such as Tanh and ReLU. The size of the networks—the number of hidden layers and the number of neurons per layer—is an important parameter to choose. If the network is too small, then it will have a very limited functional representation, resulting in a flow too smooth to fit the SV data. If it is too large, then it could result in flows that fit the SV but have spurious small-scale features. We, therefore, choose the optimal size of network through sampling in model space, finding the size that produces the minimum SV residual but has no spurious small-scale flows, through the trade-off method detailed in Shakespeare-Rees Naomi et al. (2025), which we briefly describe here. We determine the optimal network size by balancing the quality of data fit against the complexity of the resulting flow, without applying explicit regularisation. To identify suitable configurations, we evaluate networks under two regions: the Atlantic, where simple westward flow is expected (Holme 2007), and the South China Sea, which exhibits more complex, divergent flow behaviour (Whaler et al. 2022). For each network architecture, we compute the SV root mean squared error (RMSE) and the complexity as measured by the average of the squared second spatial derivative of the flow (Bloxham 1988), evaluated on the SV data grid, repeating the process across five random seeds to assess variability and robustness. These results are then visualised as trade-off curves, from which we identify the ‘knee’ point—representing the best compromise between accuracy and simplicity. Due to the relatively small size of the dataset, cross-validation was not employed, as it would require partitioning the data during training and risk degrading model performance. Using this method, we found an optimal size of 10 layers of 150 neurons. Although the PINN methodology allows us to apply additional physics-based flow constraints, we chose not to do this and to present the simplest possible model approach for this submission. In addition, while we do not explicitly apply smoothing to the flows, there is smoothing inherent to the modelling approach due to representing the flow using a finite network as specified above.

### Training data

The training procedure for the PINN methodology requires values for the SV, as well as values for the horizontal spatial derivatives of the main field. We use the core field component of the geomagnetic field model CHAOS−7.18, an extension of the CHAOS-7 model presented in Finlay et al. (2020), projected onto a grid in spherical coordinates. CHAOS−7.18 spans from January 1999 to June 2024, and is built from a combination of satellite and ground-based observatory data. This was deemed a suitable choice of model, as it has been used in multiple core flow inversion studies (eg. Gillet et al. (2022)). CHAOS−7.18 is temporally regularised to reduce non-uniqueness by penalising the second time derivative at the endpoints, and the third time derivative throughout. The time-dependent internal field model is defined up to spherical harmonic degree 20, but only the coefficients for the main field and SV up to degree 8 are used for the SV forecast, this being the degree to which the SV forecast is required in IGRF. Truncation of the secular variation at higher spherical harmonic degrees was explored, but it did not lead to improved performance in hindcasts.

**Fig. 2:**
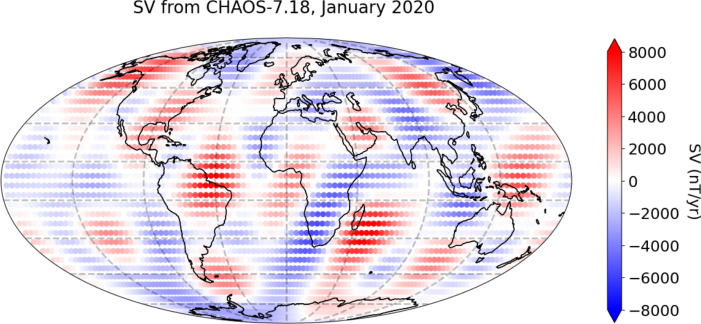
SV ($\partial B_r/\partial t$) from CHAOS−7.18 at epoch 2020.0 (Finlay et al. (2020)) at the CMB, truncated at degree 8, with continents shown for reference. Grey lines indicate the edges of the regional latitude–longitude boxes after border removal

**Fig. 3:**
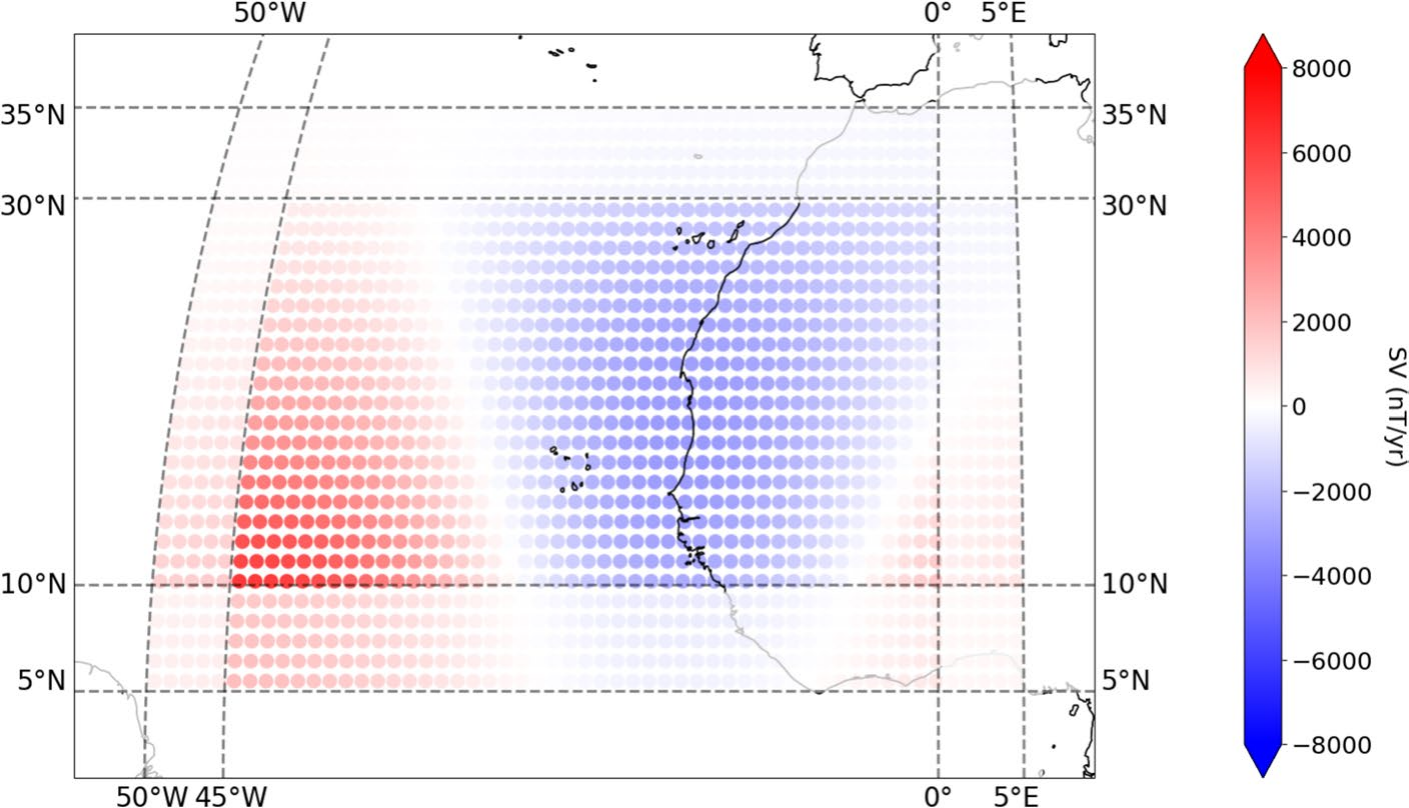
Example of a box used for inversion. After training, a 5$^\circ$ border is removed to minimise edge effects, this is shown with a transparent overlay. SV ($\partial B_r/\partial t$) from CHAOS−7.18 at $l = 8$, shown for reference. Adapted from Shakespeare-Rees Naomi et al. (2025)

We tested two approaches to core flow inversion: a ‘Regional’ method and a ‘Global’ method. For the regional approach, a mosaic of regional flow models are constructed from SV from 1st January 2020 using CHAOS−7.18. The surface of the core mantle boundary, at a radius of 3485 km, is carved up into 56 overlapping latitude–longitude boxes each spanning 30$^\circ$ by 55$^\circ$, and 16 overlapping boxes spanning 25$^\circ$ by 55$^\circ$ for the boxes closest to the poles. The boxes extend to within 5$^\circ$ of the poles. The edges of these boxes are shown by the grey lines in Fig. 2. Each of these boxes have grid points every 1$^\circ$ in both dimensions. We note that alternative partitions of the CMB, such as overlapping circular regions or tessellated triangles, are also possible, but these were considered to be beyond the scope of the present study. For each of the 72 boxes, a separate PINN is trained to find the flow that would generate the SV for that box only. The PINN for each box is trained for 5000 iterations, and results obtained for 5 seeds. The model with the lowest $L_{SV}$ (SV MSE) is taken as the ‘preferred model’ for that box. A 5$^\circ$ border is then cut off the results of each box, as in Fig. 3, with the result being 56 boxes of 20$^\circ$ by 45$^\circ$, and 16 boxes of 15$^\circ$ by 45$^\circ$. Sensitivity tests during development indicated that a 5$^\circ$ border was sufficient to maintain continuity between adjacent regions without introducing notable artefacts. Increasing the border produced only marginal improvements in transition smoothness, with no significant changes in overall flow patterns or fine-scale features, even when the border size was doubled. The remainder of each box are stitched together, with no continuity conditions imposed at the edges of the boxes. For use in the prediction methodology, the $\boldsymbol{u}$ and $\nabla \cdot \boldsymbol{u}$ values were interpolated from the training grid, using cubic spline interpolation onto the same Gauss–Legendre–Fourier grid as the advection method in Sect. 3, as this gave better hindcast results than simply evaluating the model directly onto the Gauss–Legendre–Fourier grid.

For the global flow inversion model, we projected the field coefficients of CHAOS−7.18 on one large latitude–longitude box covering most of the CMB, aiming to compare a model incorporating all available spatial data, with our regional model incorporating the same data in regional boxes. The box extends to within 5$^\circ$ of the poles, and from -180$^\circ$ to 180$^\circ$ in longitude. We do not assume any regularity conditions for this approach, and we do not impose periodicity between the longitudinal edges of the box. CHAOS−7.18 is projected onto the same Gauss–Legendre grid as the regional inversion. We did not add a border to the edges for the global flow inversion, as they would have extended over the poles.

To optimise model performance and reduce training time, the CHAOS−7.18 input and outputs of the network are re-scaled, so that their magnitudes are all about 1 (Sola and Sevilla 1997). This is achieved by expressing $B_r$ in $\mu$T, time in 0.1 yr, and length in km. Typical values of $B_r$ at the CMB are 500$\mu$T and so typical values of SV are then 1 $\mu$T/0.1 yr in these units and flow speeds are typically 1 km/0.1 yr.

## Advection methodology

Once we have found a flow model consistent with the SV for a particular year, we predict the SV assuming advection from a steady flow. We evolve the frozen-flux induction equation over timesteps of 1 year. To do this, we require a flow and an estimate of the main field at the beginning epoch. For each yearly time step $t_i$ through this period, the main field was evolved using:

7 $$\begin{aligned} \begin{aligned} \boldsymbol{B}_{t_i+1} = \boldsymbol{B}_{t_i} + \frac{\partial B}{\partial t} \bigg |_{t_i}, \end{aligned} \end{aligned}$$

where the subscripts ${t_i}$ indicate evaluation at time *i*. Numerically, we evaluate the SV using the PINN machinery on Gauss–Legendre–Fourier grid, and transform to find the component of spherical harmonic degree 8. The required horizontal field derivatives are found by projecting the field onto spherical harmonics and taking the derivatives in space. No explicit additional flow smoothing, either spatially or temporally, are used in this SV prediction. The mean of these SV snapshots is then determined by summing together each SV snapshot and then dividing by 5, such that:

8 $$\begin{aligned} \begin{aligned} \left( \frac{\partial B_r}{\partial t}\right) _{Average} = \frac{1}{5} \sum ^{5}_{i=1} \frac{\partial B}{\partial t}\bigg |_{t_i} . \end{aligned} \end{aligned}$$

## Hindcasting and comparison to IGRF-13

### Regional and global models

To evaluate our methodology against the performance of the IGRF-13 secular variation (SV) prediction candidates, we conducted a 4-year hindcast beginning on 1 January 2020 through to 1 January 2024, and compared the results with time-dependent SV from CHAOS−7.18. Only geomagnetic data from 2020 was used for our hindcasts.

**Fig. 4:**
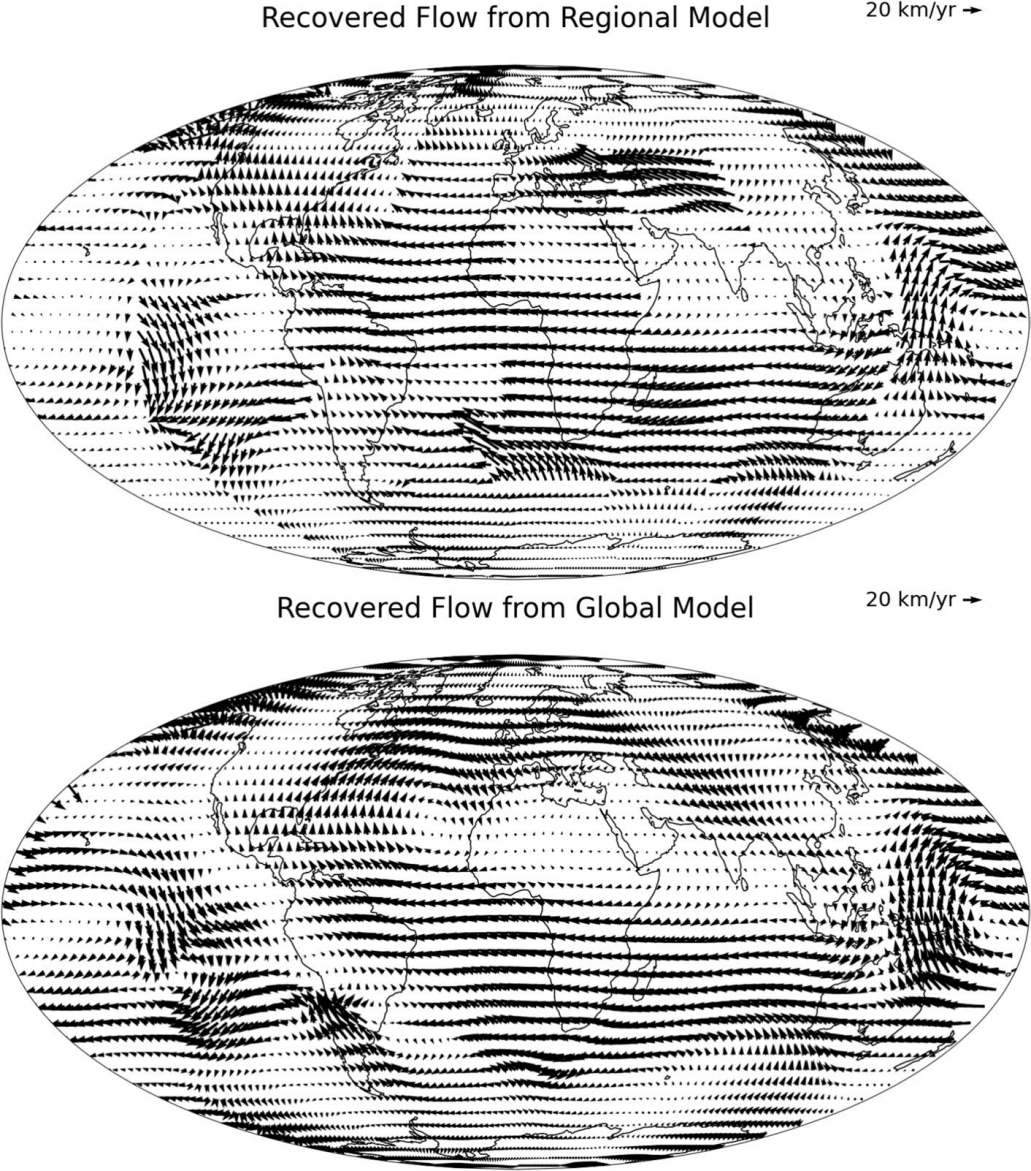
Regional (top) and global (bottom) flow models, using the PINNs inversion methodology. All plots are at the CMB. Continents shown for reference

The global Mean Absolute Error (MAE) between the SV from the CHAOS−7.18 model and the SV output from the PINN, both at epoch 2020.0, is 52 nT/year, a relative absolute error of 3.2%. The recovered flows for the regional model are shown in Fig. 4. The flows show similar large scale features as those found using global methodologies, demonstrating westward flow under the Atlantic, eastward flow under the Pacific, and the presence of the anticyclonic planetary gyre (Pais and Jault 2008; Gillet et al. 2022; Finlay 2019).

For the global approach, we trained a separate PINN for one latitude–longitude box encompassing the non-polar CMB. Again, the PINN for the box is trained for 5000 iterations, and results obtained for 5 seeds. The model with the lowest $L_{SV}$ (SV MSE) is taken as the ‘preferred model’ for the global box. The MAE between the SV from the CHAOS−7.18 model and the SV output from the PINN is 15 nT/year, a relative L1 error of 0.8%. The recovered flows for the global model are shown in the bottom panel of Fig. 4. The flows from the global model are smoother than that of the regional model, and have reduced small scale features. There are also sections of southerly flow under Asia, which is not present in the regional model.

**Fig. 5:**
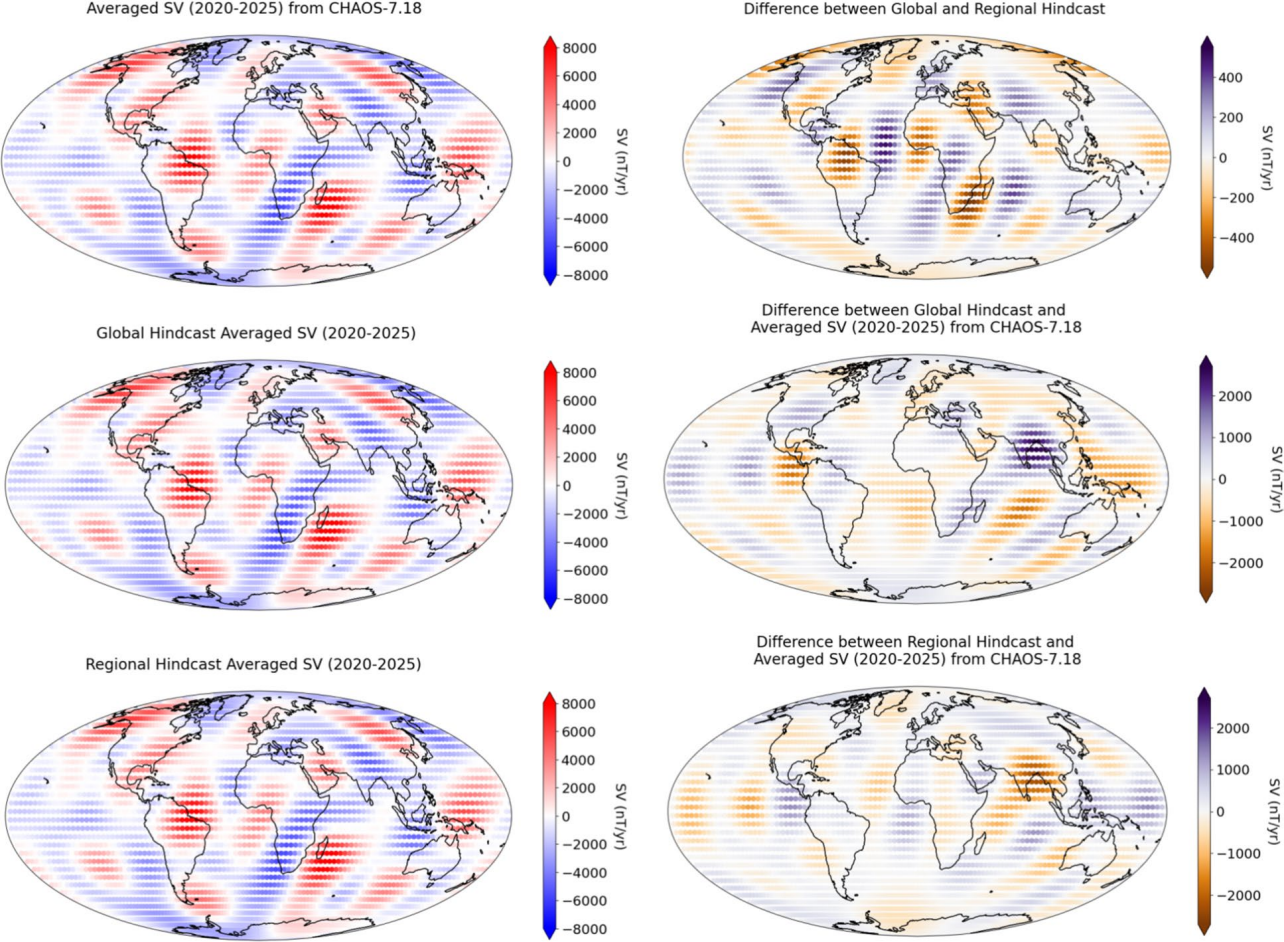
Top right panel: Average SV ($\partial B_r/\partial t$) over the period 2020–2025 from the CHAOS−7.18 model. Middle right panel: Hindcast averaged SV ($\partial B_r/\partial t$) (2020–2025) using the ‘Global’ flow model. Bottom right panel: Hindcast averaged SV ($\partial B_r/\partial t$) (2020–2025) using the ‘Regional’ flow model. Top left panel: Absolute difference between the ‘Global’ and ‘Regional’ hindcasts. Middle left panel: Absolute difference between the ‘Global’ hindcast and the average SV ($\partial B_r/\partial t$) over the period 2020–2025 from the CHAOS−7.18 model. Bottom left panel: Absolute difference between the ‘Regional’ hindcast and the average SV ($\partial B_r/\partial t$) over the period 2020–2025 from the CHAOS−7.18 model. All plots are at the CMB. Continents shown for reference

Both the global and regional flows are then used in the advection methodology detailed in Sect. 3, to produce global and regional hindcasts. The absolute difference between the global and the regional hindcasts at the CMB are shown in Fig. 5. The maximum difference in SV hindcast is -400nT/year, largely concentrated underneath South America, with other areas of increased difference, including the northern hemisphere, close to the location of the high latitude jet presented in Livermore et al. (2017). The areas of largest difference correspond to the areas of largest flow difference between the two flow models, which is a result of increased small scale flow.

**Fig. 6:**
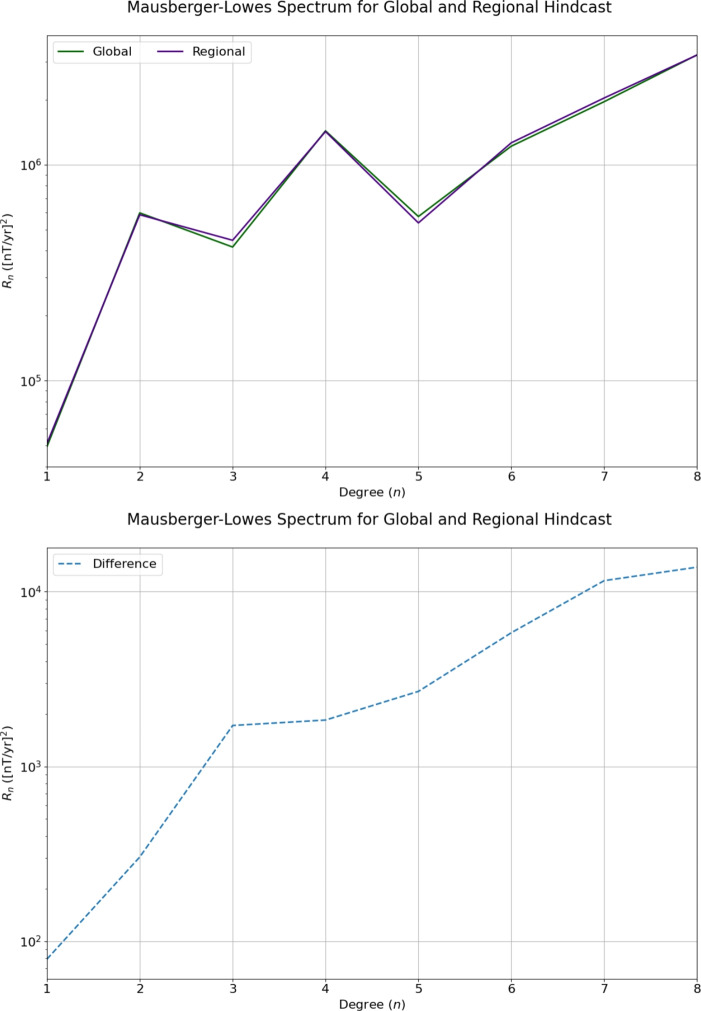
Top panel: Mausberger–Lowes spectrum of the SV for the global and regional hindcasts. Bottom panel: Difference between the global and regional models. The two spectra are exceedingly similar, having a relative L1 difference of 1.8% relative to the regional model

To further investigate the difference between the global and regional hindcast, the Mausberger–Lowes spectrum of the SV hindcasts was plotted for each model at the Earth’s surface. This is shown in Fig. 6, with the difference between them indicated by the dashed line. These models are almost identical, having a relative L1 difference of 1.8% relative to the regional model at the Earth’s surface, indicating that considering one large grid or many smaller grids are very similar, provided the solutions are converged. The relative L1 difference becomes 1.7% relative to the regional model at the CMB.

### Comparison to IGRF-13

To compare our hindcasting models to the IGRF-13 SV candidate models, as well as the averaged SV from CHAOS−7.18 between 2020 and 2024, we first plot the Mausberger–Lowes spectrum of the SV at the Earth’s surface for all of the considered models. The candidate models of IGRF-13 are displayed using dashed lines, the global and regional models presented in this study are displayed using purple and dark green, respectively, and the spectra from the average SV between 2020 and 2024 is shown with a solid black line.

**Fig. 7:**
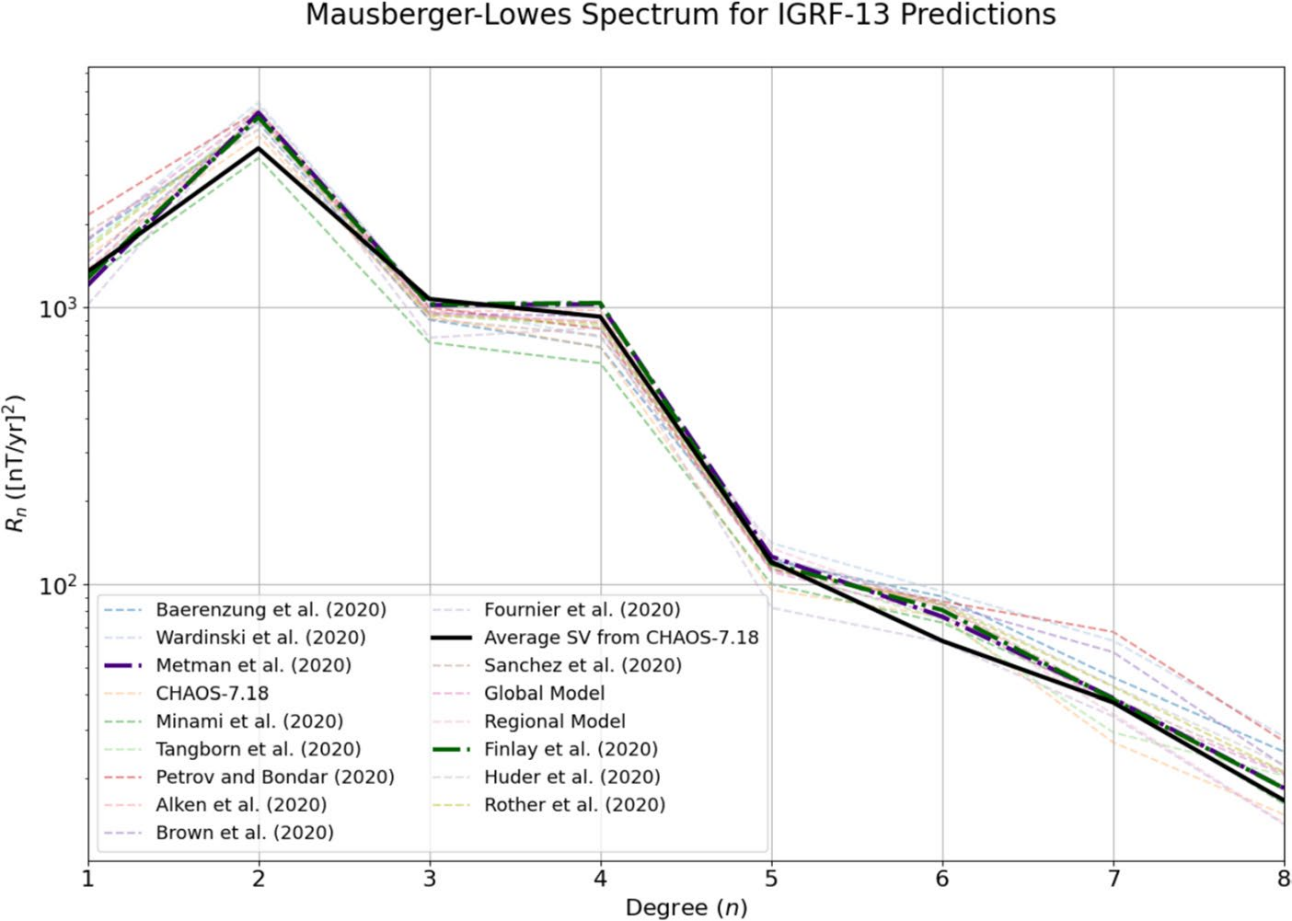
Mausberger–Lowes spectrum for our global and regional hindcasts, as well as the other IGRF-13 candidate models. The inferred Mausberger–Lowes spectrum from the average SV between 2020 and 2025 from the CHAOS−7.18 model is marked by ‘Average SV from CHAOS−7.18’

Figure 7 shows that the majority of candidate models overestimate the energy at degrees 1–2 and 6–7, whereas at degree 3–5 the majority of models underestimate the energy. Conversely, both of our models slightly underestimate the degree 1 contribution to the SV, then overestimate the degree 4–8 contributions, with the regional model contributing more at degree 6 than the global model. It is perhaps expected that the regional model contributes more to the SV at higher degrees than the global model, due to the presence of increased small-scale flow, increasing the complexity of the model. Reassuringly, all of these models are similar to the observed SV, particularly at larger scales, though our models follow the black line more closely than many of the other models.

To further probe the hindcast performance, we evolved the main field between 2020.0−2024.0, from an initial state at 2020 derived from CHAOS−7.18, using each of the candidate average SV predictions and our own, such that for each year, and each candidate:

9 $$\begin{aligned} \begin{aligned} \boldsymbol{B}_{t_i+1} = \boldsymbol{B}_{t_i} + \frac{\partial \boldsymbol{B}}{\partial t}\bigg |_{Average}. \end{aligned} \end{aligned}$$

The Root Mean Square (RMS) of the difference between the predicted $B_r$ and the modelled $B_r$ from CHAOS−7.18, at degree 8 and at each timestep, was then calculated for each candidate both at the CMB and the Earth’s surface.

**Fig. 8:**
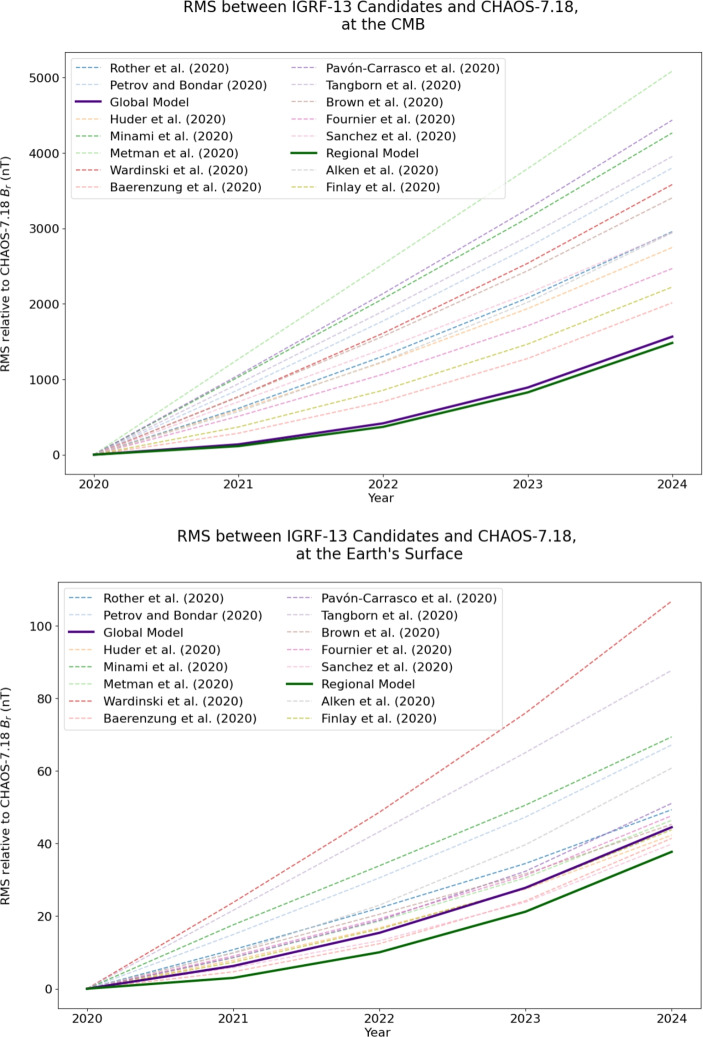
RMS between IGRF-13 candidates and CHAOS−7.18 derived $B_r$ values over time. The regional model performs marginally better than the global model, with the difference becoming more pronounced over time

Figure 8 (top panel) shows that both of our models have reduced RMS compared to the other IGRF candidates, at the CMB, with the regional model performing slightly better than the global model. To ensure the robustness of this result and confirm it was not an artifact of our model choice, we compared the predicted SV from flows trained on CHAOS−7.18 with SV from the Kalmag model (Baerenzung et al. 2020). In this comparison, both the global and regional models continued to show reduced RMS difference compared to the other candidate models. This is shown in the figures in Appendix B. The bottom panel of Fig. 8 shows the results at the Earth’s surface, relative to CHAOS−7.18. Once again, our regional model demonstrates reduced RMS differences compared the other models in this hindcast. However, our global model is outperformed by the models from the Max Planck Institute (Sanchez et al. 2020), IPGP (Fournier et al. 2021), Potsdam (Baerenzung et al. 2020) and DTU (Finlay et al. 2020). Therefore, it seems that the small-scale flows present in the regional modelling contribute to the SV in a way that improves forecast performance.

## Candidate SV model

**Fig. 9:**
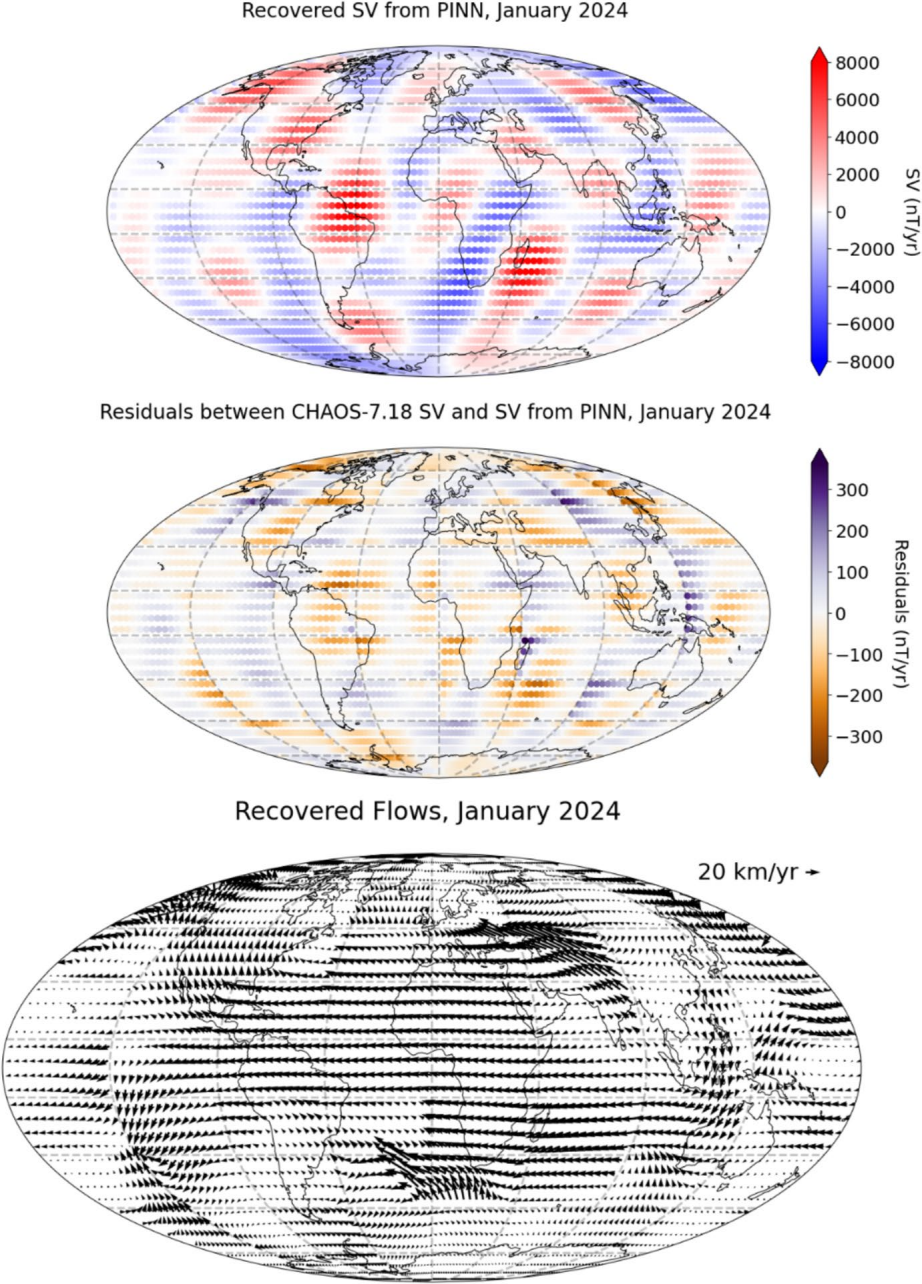
Top panel: Recovered SV using the horizontal flows in January 2024, with the box borders shown using the grey dashed lines. Middle panel: Residual between CHAOS−7.18 SV and the Recovered SV. Bottom panel: Recovered Flows from the PINN. The flows show large-scale features found in prior studies, such as westward drift under Atlantic, eastward flow under Indonesia, and the eccentric planetary gyre. Continents shown for reference. The flow at epoch 2024 is assumed to be the same as for 2025 for the purposes of SV prediction

With the hindcast performance of the regional model in mind, we chose this methodology to determine the flows used in our steady-flow prediction methodology. We used the same methodology as the hindcast, but using the CHAOS−7.18 geomagnetic field data from 1st January 2024. The radial magnetic field at 2025 was estimated by adding the SV on 1st January 2024 to the radial magnetic field at the same date, in the same manner as in Eq. (9), with $\text{ d }t = 1$ year, to predict the field in 2025. Ideally we would use the field in 2025, but this is outside the timeframe of CHAOS−7.18. The recovered SV from the PINN, and the residual between the CHAOS−7.18 SV and the recovered SV, are shown in the top panels of Fig. 9. At the epoch for which the PINN is trained, the recovered SV agrees well with the training SV, as the MAE between the recovered SV and CHAOS SV is 50 nT/year, a relative L1 error of 3.2%. This is similar to the hindcast values, giving hope that the forecast may fair similarly well. The recovered flows are shown in the bottom panel of Fig. 9, again showing westward drift under Atlantic, eastward flow under Indonesia, and the eccentric planetary gyre.

**Fig. 10:**
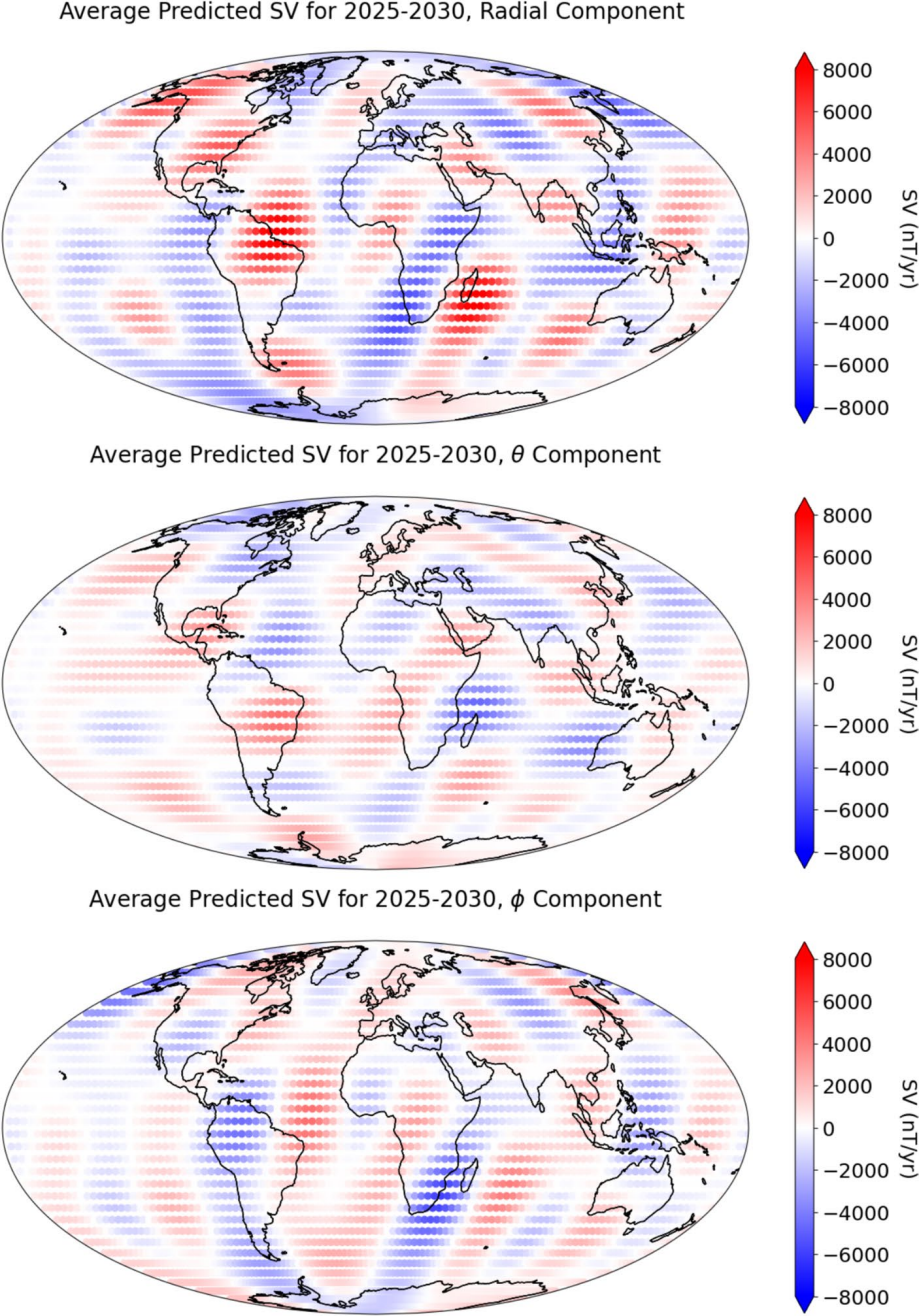
Predicted SV (top: $\partial B_r/\partial t$, middle: $\partial B_{\theta }/\partial t$, bottom: $\partial B_{\phi }/\partial t$) for epochs 2025.0−2030.0 at the core–mantle boundary. Continents shown for reference

We then advect the magnetic field, assuming the flow is steady and using the estimated values of the radial magnetic field at 2025. We note that due to a data transmission error at this step, the original University of Leeds SV prediction candidate for IGRF-14 submitted in October 2024 was incorrect. We show the corrected University of Leeds SV prediction candidate for 2025–2030 in Fig. 10, and provide the updated Gauss coefficients in Appendix A.

## Conclusions and discussion

We present a methodology for SV forecasting, using Physics-Informed Neural Networks to determine outer core flows for use in a steady-flow advection method to predict SV for IGRF-14. To investigate the performance of the method, we produce a hindcast for the epochs 2020.0−2025.0, using a mosaic of regional flows (‘Regional Model’) and a global flow (‘Global Model’).

We then compared our hindcasts to other IGRF-13 SV forecast candidate models, as well as to the average CHAOS−7.18 SV between epochs 2020–2025. It should be noted that the average SV during this time period—what is predicted in our hindcasts and IGRF-13 candidate models—may differ from CHAOS−7.18 SV observations, as un-modelled processes in the outer core leading to variable SV change over time, such as the presence of geomagnetic jerks (Brown et al. 1957; Aubert et al. 2022). The results show that the regional models outperformed the global model and the other IGRF-13 candidates, both at the CMB and at the Earth’s surface.

There are a number of considerations and potential improvements that need to be accounted for when evaluating our models. The first is the box size defining both regional and global flows, along with the (removed) additional border. While the border removal approach works well for the regional boxes, acting to mitigate any edge effects, these edge effects are still seen in the global model. Adding a border region did not act to prevent this, and so further work may include altering the global methodology to remove this, as has been done in Jinfeng et al. (2024). The use of MSE for in our loss function may also affect this, as when the loss is minimised the optimiser will prioritise a fit over the bulk of the box, rather than the edges which comprise only a small part.

The second consideration is our choice of geomagnetic field model for use as training data. We chose CHAOS−7.18 for the input field model, due to its use in multiple core inversion studies, such as Gillet et al. (2022) and Finlay (2019). Other geomagnetic field models such as Kalmag (Baerenzung et al. 2020) also covered the time frame considered in this study, and the use of this model as our training data may have yielded slightly different results. However, the high quality data from INTERMAGNET (Love and Chulliat 2013) and Swarm mean that these field models are well-constrained, such that there is little difference between Kalmag and CHAOS. Since the publication of IGRF-14, the 8th generation of CHAOS has been released (Kloss et al. 2024). CHAOS-8 is temporally regularised by taking temporal covariances from geodynamo simulations, whereas CHAOS-7 is temporally regularised by penalising the second time derivative of the radial field over the CMB at the endpoints, and its third time derivative throughout. This change in regularisation method influences the small-scale features of the SV, which may, in turn, impact the recovered flow patterns. Further work might include incorporating this model into our prediction methodology, to see whether these small scale features increase the forecasting abilities of our model.

Third, while previous studies involving the use of PINNs for core flow inversions have applied the Tangential Geostrophy flow constraint (Jinfeng et al. 2024; Shakespeare-Rees Naomi et al. 2025), in this modelling approach we chose not to apply a flow constraint, due to the complexities of balancing this constraint in the loss function. Subsequent work may include quantifying the effect of different flow assumptions on forecasting ability.

Finally, we note that the regional models are first trained on grids equivalent to 1 point per spatial degree, and then interpolated to the same Gauss–Legendre–Fourier grid that the global methodology is trained on. Further work will need to be done to investigate whether the enhanced performance of the regional methodology is due to the difference in training grid, or if the regional aspect of the model provides information that aids the forecast.

Our regional Physics-Informed Neural Network approach to forecasting secular variation demonstrates slightly improved performance over previous generations of SV forecasts in hindcasting experiments, though it remains to be seen how our model performs in the next 5 years. As the error between the predicted and ’true’ $B_r$ increases with time (Fig. 8), future work could explore whether weighting earlier time steps more strongly in Eq. (8) improves forecasting performance. With ongoing advances in machine learning, forecasting methodologies that leverage these techniques will become increasingly prevalent in geomagnetic research. The integration of regional methodologies—such as the one presented in this study—with the additional coverage provided by low-inclination satellite missions such as MSS-1 (Jiang et al. 2024) and NanoMagSat (Deconinck et al. 2024) holds particular promise for enhancing the accuracy of future secular variation forecasts, given the similarly regional nature of the data they provide. Further additions to this methodology could include incorporating time-dependent constraints, leveraging the full CHAOS−7.18 time series. Localised estimates, such as those from virtual observatories (Mandea 2006; Hammer et al. 2021) or from SOLA (Subtractive Optimally Localised Averages, Hammer and Finlay (2018)), could also be trialled, ensuring there is no global constraint at any stage. Since conventional global field and flow modelling techniques work with purely regional data, the development of robust regional approaches is essential—not only to fully leverage upcoming missions, but also as a necessary redundancy should Swarm fail, or if NanoMagSat launches without its planned polar-orbiting component.

## Summary

This study presents a novel methodology to forecast SV, using flows from a physics-informed neural network-based approach in a steady-flow advection scheme. Two approaches were presented: an approach comprising a mosaic of regional flow inversions and a single global flow inversion. Hindcasting results reveal that our regional approach outperforms the IGRF-13 SV candidate models, both at the CMB and the Earth’s surface. We present our updated candidate SV model over the time period 2025–2030 for IGRF-14, derived from advected flows from the regional inversion approach.

## Figures and Tables

**Fig. 11:**
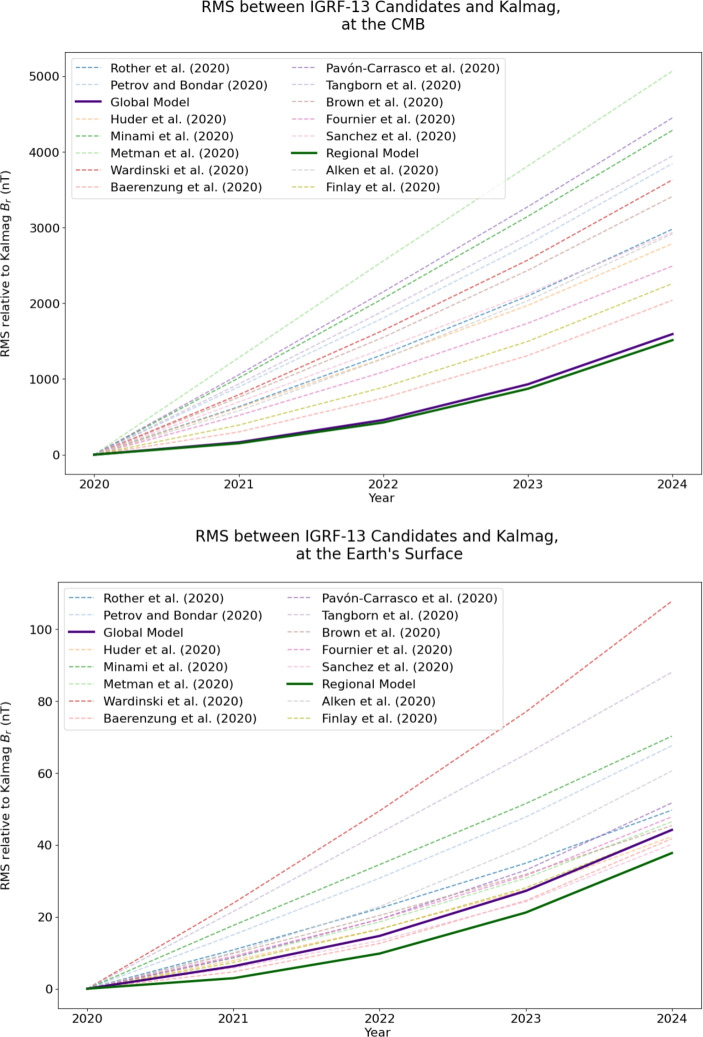
RMS between IGRF-13 candidates and Kalmag derived $B_r$ values over time. The regional model performs marginally better than the global model, with the difference becoming more pronounced over time

## Data Availability

Example code, as well as updated SV forecast gauss coefficients in .cof format, can be found here: (https://github.com/geonaomi/LeedsIGRF/blob/main/. The CHAOS-7 model can be accessed at: https://www.spacecenter.dk/files/magnetic-models/CHAOS-7. The IGRF-13 candidate models can be found here:https://github.com/IAGA-VMOD/IGRF13eval/tree/main/data.

## Appendix A: updated gauss coefficients for leeds’ candidate SV model

See Table 1

**Table 1:** Updated Gauss coefficients for the University of Leeds IGRF Candidate model

*n*	*m*	$\dot{g}_{nm}$	$\dot{h}_{nm}$
1	0	14.47	0.0
1	1	9.38	−22.10
2	0	−11.30	0.0
2	1	−5.24	−27.36
2	2	−8.75	−11.15
3	0	−2.02	0.0
3	1	−4.47	3.82
3	2	0.69	−0.19
3	3	−16.12	−4.11
4	0	−1.61	0.0
4	1	−2.48	−1.21
4	2	−5.92	4.10
4	3	5.58	1.40
4	4	−7.02	−4.0
5	0	0.64	0.0
5	1	1.32	−0.58
5	2	0.14	2.17
5	3	0.56	0.51
5	4	2.47	1.49
5	5	0.79	2.13
6	0	−0.13	0.0
6	1	−0.46	0.35
6	2	0.85	−1.50
6	3	1.17	−0.34
6	4	−0.82	0.87
6	5	0.41	0.72
6	6	0.93	0.94
7	0	−0.25	0.0
7	1	−0.06	0.64
7	2	−0.12	0.49
7	3	0.47	−0.76
7	4	−0.12	0.11
7	5	−0.78	−0.97
7	6	−0.81	0.63
7	7	0.92	−0.25
8	0	−0.13	0.0
8	1	0.22	−0.25
8	2	0.04	0.45
8	3	0.49	−0.41
8	4	−0.14	0.39
8	5	0.25	−0.57
8	6	0.062	−0.68
8	7	−0.039	0.28
8	8	0.22	0.23

These coefficients can be found in .cof format here: (https://github.com/geonaomi/LeedsIGRF/blob/main/Leeds_IGRF14_update.cof)

## Appendix B: Comparison between IGRF-13 candidates and Kalmag

See Fig. 11

## Abbreviations

SV: Secular variation
IGRF: International Geomagnetic Reference Field
IAGA: International Association of Geomagnetism and Aeronomy
CMB: Core Mantle Boundary
PINNS: Physics-informed neural networks
NN: Neural networks
FC-NN: Fully connected neural networks
MSE: Mean square error
MAE: Mean absolute error
RMS: Root mean square error
LOD: Length of day
